# A Closer Look at Estrogen Receptor Mutations in Breast Cancer and Their Implications for Estrogen and Antiestrogen Responses

**DOI:** 10.3390/ijms22020756

**Published:** 2021-01-13

**Authors:** Léa Clusan, Pascale Le Goff, Gilles Flouriot, Farzad Pakdel

**Affiliations:** Inserm, EHESP, Irset (Institut de Recherche en Santé, Environnement et Travail)-UMR_S1085, Rennes University, F-35000 Rennes, France; lea.clusan@univ-rennes1.fr (L.C.); pascale.le-goff@univ-rennes1.fr (P.L.G.); gilles.flouriot@univ-rennes1.fr (G.F.)

**Keywords:** estrogen receptor, breast cancer, endocrine resistance, mutation, receptor folding

## Abstract

Breast cancer (BC) is the most common cancer among women worldwide. More than 70% of BC cases express estrogen receptor alpha (ERα), a central transcription factor that stimulates the proliferation of breast cancer cells, usually in the presence of estrogen. While most cases of ER-positive BC initially respond to antiestrogen therapies, a high percentage of cases develop resistance to treatment over time. The recent discovery of mutated forms of ERα that result in constitutively active forms of the receptor in the metastatic-resistance stage of BC has provided a strong rationale for the development of new antiestrogens. These molecules targeting clinically relevant ERα mutants and a combination with other pharmacological inhibitors of specific pathways may constitute alternative treatments to improve clinical practice in the fight against metastatic-resistant ER-positive BC. In this review, we summarize the latest advances regarding the particular involvement of point mutations of ERα in endocrine resistance. We also discuss the involvement of synonymous ERα mutations with respect to co-translational folding of the receptor and ribosome biogenesis in breast carcinogenesis.

## 1. Introduction

### 1.1. Breast Cancer Types

Millions of women develop breast cancer worldwide, representing a major health issue. Notably, breast cancer also exists in men but is very rare, accounting for fewer than one percent of cases. This disease generally arises from the proliferation of epithelial cells in the lobules or lactiferous ducts of the mammary gland and is a very heterogeneous malignancy. According to histopathological data, breast cancers are classified as lobular or ductal, in situ or invasive carcinomas preferentially colonizing bone, liver, lung or brain. In addition to histological grade and cancer stage determination, the development of molecular techniques has shed light on the heterogeneity of molecular profiles and gene expression across the types of breast cancer, resulting in more than 20 subtypes of breast carcinoma being characterized [1,2]. Molecular markers rely primarily on the expression of relevant receptors, including estrogen receptor alpha (ERα), progesterone receptor (PR) and human epidermal growth factor receptor 2 (HER2). Various expressions of these receptors by breast cancer cells are correlated with different degrees of differentiation and aggressiveness of the tumor. Knowing these characteristics enables improved prognosis and selection of the most relevant therapy [3,4]. The simplest classification relies on three major subtypes: luminal, HER2-enriched, and triple-negative, as shown in Table 1 [5].

The luminal type of breast cancer is divided into two subclasses: A and B. Both are characterized by the expression of ERα, but differ in terms of aggressiveness: luminal A breast cancers are usually low-grade, whereas luminal B cancers display overexpression of HER2, reduced ERα expression, and increased proliferation. Such breast cancers are predominant, especially luminal A, and convey a better prognosis because these tumors depend on estrogen for their growth, and specifically targeting estrogen through endocrine therapy to block its proliferative action, which is an effective strategy. As reviewed by Jensen and Jordan, the identification of the estrogen receptor and subsequent understanding of its implication in breast cancer paved the way for developing targeted therapy [8]. The selective estrogen receptor modulator (SERM) tamoxifen was first developed during the 1970s and became the standard of care for breast cancer, as it enabled the saving of many lives with fewer side effects than chemotherapy. Nevertheless, the use of tamoxifen presents some drawbacks, which led to the development of additional SERMs, as reviewed by Maximov et al. [9]. While SERMs are competitive inhibitors of ERα that prevent its activation by estrogens, another way to counteract ERα activity relies on using selective estrogen receptor downregulators (SERDs), whose binding to ERα results in the degradation of the receptor. Finally, another therapeutic approach aims to directly deprive the tumor of estrogen by ovariectomy or the use of aromatase inhibitors. The development of this class of agents began during the 1980s by Brodie and colleagues and had benefits for patients who do not respond to SERMs [10]. However, for 30–50% of ER-positive breast cancers, resistance to endocrine therapy occurs. For this large number of patients, the prognosis is worse, which raises real public health concerns [5].

### 1.2. ERα Activity

Estrogen receptors are nuclear receptors that mediate estrogen actions by regulating gene expression. Two highly homologous protein isoforms exist in vertebrates, ERα and ERβ, encoded by two independent genes. However, several primarily in vivo studies have shown that their activity differs in mammary gland development and nonreproductive tissue functioning, as well as in breast cancer pathogenesis [11]. This difference is in line with their differential tissue expression. Concerning breast cancer, clinicopathological data notably reveal that ERβ levels decrease during carcinogenesis [12,13]. The precise role of ERβ remains elusive; several lines of evidence confer it tumor suppressive activity, but further studies are needed to gain insights into the mechanisms involved [14].

**Figure 1:**
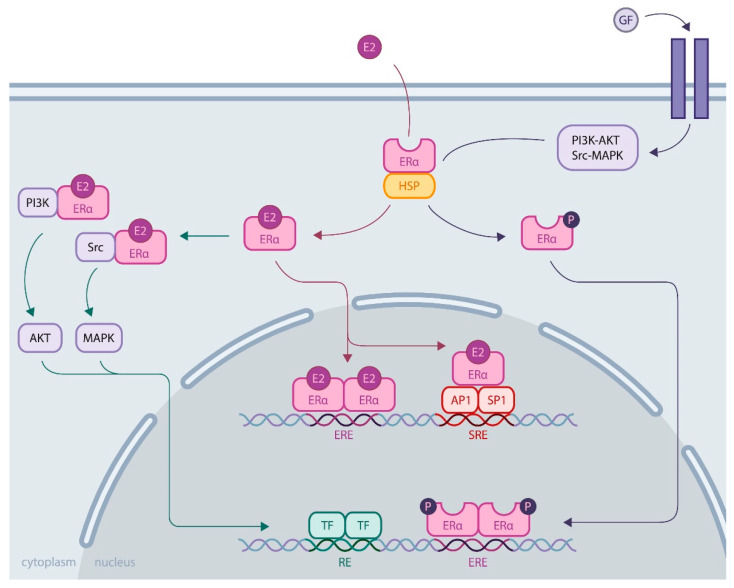
ERα activity through genomic and nongenomic actions. **Genomic activity**: ERα activation upon estrogen binding (E2) or after its phosphorylation by cellular kinases following growth factor (GF) receptor stimulation allows its release from heat shock proteins (HSPs). Then, ERα translocates into the nucleus, where it binds DNA by direct (through estrogen responsive elements, EREs) or indirect mechanisms (upon binding to other transcription factors, such as AP1 or SP1, that bind DNA through serum responsive elements, SREs). **Nongenomic activity**: Activated ERα interacts with cellular kinases (e.g., PI3K and Src), leading to signaling pathway stimulation involving AKT or MAPK, for example, eventually resulting in transcription factor (TF) activation. All these mechanisms induce transcriptional activation or repression of the regulation of cell fate.

Much more is known about ERα. Its activity is essential for the reproductive system, and its expression is increased in most breast cancers, which has led to extensive research to understand its regulatory role. ERα is a ligand-inducible transcriptional factor. After ligand binding and dimerization, ERα is recruited to the promoter region of the target genes, either by binding, directly targeting DNA sequences called EREs (estrogen responsive elements), or by protein/protein interactions with other transcriptional factors, such as AP1 or SP1. Recent development of chromatin immunoprecipitation of DNA coupled to high-throughput sequencing (ChIP-Seq) techniques has led to the identification of the ERα cistrome in mammary adenoma carcinoma cell lines, such as MCF-7, with 5000 to 10,000 estrogen receptor binding sites (ERBS), three quarters of which are EREs [15]. This results in the recruitment of numerous coactivators (members of the p160 family, CBP/P300, members of the SWI/SNF family, members of the mediator complex, etc.) via the AF1 and AF2 transactivation domains of ERα in an ordered, cyclic and combinatorial process, leading to transcriptional activation of target genes [16]. In addition to this activity at the genomic level, ERα also has nongenomic activity by interacting in the cytoplasm with cellular kinases that activate various signaling pathways, such as PI3K-AKT and Src-MAPK (Figure 1). These rapid actions of the receptor may ultimately result in the regulation of gene expression, highlighting the complex interrelationships between membrane and nuclear-induced events. All these modes of action ultimately lead to the regulation of cell fate, resulting in a balance between proliferation, differentiation and cell survival [11].

### 1.3. Role of ERα in Breast Cancer

Many players are involved in breast carcinogenesis at the cellular level. Notable examples include tumor suppressor genes BRCA1/2, TP53 or PTEN undergoing loss-of-function mutations or decreased expression, receptor tyrosine kinases overexpression, such as EGFR, IGF1R or HER2, whose downstream signaling pathways promote cell proliferation and invasion (PI3K-AKT, RAS-MAPK, JNK) or overexpression of the oncogene c-Myc [17,18].

However, for most breast cancers, ER deregulation plays a major role, participating in aberrant cell proliferation and leading to tumor development. This deregulation of ERα function is due to multiple phenomena, implicating a shift in the balance of cofactors in favor of coactivators, the overexpression and function of growth factor receptors whose signaling pathways (MAPK, PI3K, etc.) result in ERα activation or alteration of ERα expression at the genetic level through epigenetic regulation [19,20,21]. ERα can also be mutated and become constitutively active, but it is not the primary source of breast cancer development. Instead, activating mutations are acquired following estrogen deprivation therapies as a resistance mechanism of tumor cells to escape hormonal control and promote cell proliferation through ligand-independent activation of ERα [22]. Breast cancer cells have evolved into many other strategies against endocrine therapies, as reviewed by Musgrove and Sutherland [23].

Recent developments in biophysical techniques have enabled us to gain insights into ERα conformational changes related to mutations, causing increasing interest for understanding of the ERα response to estrogen and antiestrogens. Whereas several ERα alterations have been reviewed elsewhere, such as gene amplification or translocation [24], as well as splice variants [22], this review aims to summarize the latest advances relative to the particular implication of ERα point mutations in endocrine resistance.

## 2. ERα Missense Mutations

### 2.1. The Ligand-Binding Domain

**Figure 2:**
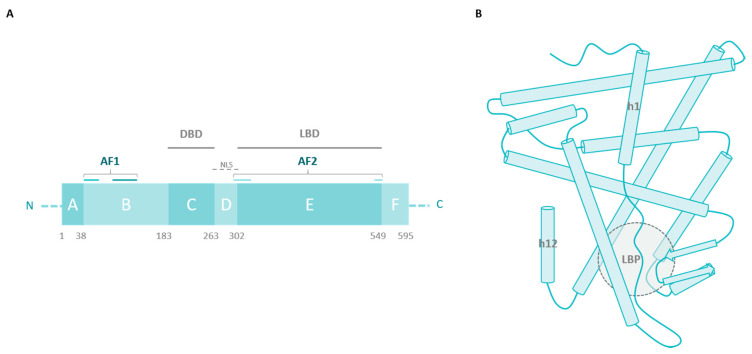
(**A**) ERα is composed of 595 amino acids forming 6 domains, from A to F. Transcriptional activation function 1 (AF1) is localized in the N-terminal region of the receptor, whereas a second transactivation function (AF2) is generated at the C-terminus when conformational rearrangements take place. The C and E domains contain the DNA- and ligand-binding domains, respectively (DBD, LBD). Finally, the D domain is called the hinge region, which participates in conformational changes and protein/protein interactions and contains nuclear localization signals (NLS) [11]. (**B**) A schematic representation of ERα LBD (from the crystal structure 1GWR deposited in the Protein Data Bank) with the ligand-binding pocket (LBP) depicted.

Since the first discovery of a missense mutation of ERα in a breast cancer sample in 1997 [25], several mutations have been identified through cohort studies. By comparing mutations detected in samples of primary versus metastatic tumors, it has been demonstrated that most missense mutations are acquired under selective pressure of endocrine therapies that create a low-estrogen environment, such as aromatase inhibitors [26,27]. In fact, an analysis performed by The Cancer Genome Atlas Network in 2012 did not detect significant mutations in the ERα gene (*ESR1*) in primary breast cancer samples contrary to other genes, such as PIK3CA (49% of luminal A tumor samples) or TP53 (32% of luminal B patients) [28]. When investigating *ESR1* mutations in metastatic breast cancer, however, the prevalence of missense mutations expands to 20–50% [29,30]. Notably, these mutations are localized in the ligand-binding domain (LBD) of ERα (Figure 2A), and several biophysical and functional studies have enabled us to decipher their consequences on ERα activity and their role in endocrine resistance.

The LBD is a highly structured region with three layers of α-helices (h1 to h12) and two β-sheets forming a hydrophobic pocket where the ligand binds (Figure 2B). Agonist binding induces structural modifications of the receptor, where h12 plays a critical role in generating a more compact conformation of ERα. These structural rearrangements participate in coactivator recruitment for ERα transcriptional activity. Antagonist binding, however, inhibits the receptor by preventing h12 from folding properly [11]. Thus, it is not surprising that most mutations acquired by ERα, in response to antiestrogens, localize in the LBD and impact ligand binding.

The most prevalent ERα mutation is a substitution of the amino acid Y537 in S, N or C (a Y537D mutation was also observed in one patient) with a prevalence reaching 60% of mutations detected in metastatic breast cancer samples [29,31]. Such mutations result in a conformational modification of the receptor that stabilizes it in its agonist form, conferring ligand-independent, constitutive activity to the mutated receptor. According to the crystal structures of the Y537S mutant, this conformational modification is due to replacement of the Y537-N348 interaction with a S537-D351 hydrogen bonding that optimizes the h11-h12 loop in the agonist conformation [32]. In this conformation, coactivators can be recruited to the AF2 cleft, and Fanning et al. showed that this binding occurs with a high affinity for the Y537S mutant, even in the absence of estrogens. This explains why this mutation confers constitutive ligand-independent activity to ERα [33]. The same study demonstrated that conformational rearrangements occurring around the h11-12 loop of the receptor, conferring an agonist-bound-like structure to ERα, reduce its affinity for the SERM tamoxifen. In addition, it was suggested that the SERM-bound Y537S mutant adopts an altered conformation compared to the wild type receptor bound to tamoxifen, participating in a decrease in efficacy of such therapeutic agents. SERDs such as fulvestrant, however, target h12 in a different way and still inhibit ERα, but increased therapeutic doses seem to be necessary [34,35]. Of note, the Y537S and C mutations were also detected in vitro in breast cancer cell lines after depriving them of estrogen to mimic the acquisition of endocrine resistance. In line with previous studies, these mutations confer ligand-independent activities to ERα and altered responses to endocrine therapy [36].

Another LBD mutation particularly observed in patients following antiestrogen treatment affects D538 (at a frequency of 20%, as reported by Katzenellenbogen et al. [32]). When substituted with G, modifications of the electrostatic environment and an increase in h12 flexibility occur, which also results in stabilization of the agonist form of ERα [32]. This leads to increased affinity for coactivators in a ligand-independent way, conferring constitutive activity to the receptor, but this activity is moderate, compared to Y537 mutations. Insights into this phenotypic difference were provided by structural studies, showing that the agonist-bound-like conformation conferred by the D538G mutation is less stable than the conformation permitted by the Y537S mutation [33]. The D538G substitution still confers increased migratory capacities to cancerous cells, which probably contribute to metastasis [37]. A study by Li and colleagues analyzed the growth of ER-positive breast tumors carrying the ER Y537S mutation in patient-derived xenografts (PDXs) after transplantation into ovariectomized mice [38]. Results showed greater tumor growth compared to tumors with wild type ERα under low estrogen conditions and an incomplete response to antiestrogenic treatments. Other studies have evaluated the response of ERα mutations (Y537S, D538G) to estradiol and antiestrogens by measuring the activation of a reporter luciferase gene [34], or endogenous ERα target genes [35]. These ERα mutants exhibited high constitutive transcriptional activation, in contrast to wild type ERα, which shows low activity in the absence of estradiol. In addition, mammary cancer MCF-7 cell lines that stably express ERα Y537S and D538G showed dramatically higher proliferation than wild type MCF-7 cells, suggesting that these ERα mutations induce a significant growth consequence in breast cancer cells [35]. Of note, Y537S and D538G mutants showed an increase in the interaction with the transcriptional coactivators AIB1 and SRC-1 compared to the wild type receptor, which is consistent with the increase in their ligand-independent activity observed in reporter gene assays [26].

Additional mutations were detected at this hotspot in breast tumor samples, L536R, P535H, V534E etc., but were less prevalent (usually less than 5% of the mutations detected in metastatic breast cancers), and studies investigating their effect on ERα conformation and crystal structures are especially lacking [30]. As those mutations also confer ligand-independent activity to ERα, it is reasonable to speculate that the mechanisms involved rely at least partially on stabilization of the receptor in its agonist form as well.

Concerning L536, for example, its replacement with R, Q, P or H probably reduces the hydrophobicity of the environment, which enables the rearrangement of the h11-h12 loop favoring the agonist conformation of ERα in the absence of estrogens; this altered conformation increases the binding of coactivators for ligand-independent activity [32].

Finally, it is noteworthy that other LBD-activating mutations are outside the mutational hotspots previously mentioned.

E380Q notably appears to be the third prevailing ERα mutation, with a detection rate up to 14% among patients with advanced breast cancer following aromatase inhibitor treatment [29]. This would neutralize charge repulsion between residues in h5 and h12, which would favor an active conformation of the receptor without ligand binding [32]. In vitro studies indeed emphasized the constitutive activity of this mutated receptor, resulting in increased target gene transcription and cell proliferation in the absence of estrogens [39,40]. Evidence is lacking to explain the underlying mechanisms because ligand-independent coactivator binding does not seem to be involved [40]. One hint could be a defect in the corepressor PHB2 binding, which would enhance ERα signaling [41]. Additionally, increased sensitivity to estrogens has been reported, which contributes to promoting tumor growth and resistance to aromatase inhibitors [39].

Finally, the S463P mutation is less documented (with an apparent distribution inferior to 4% in metastatic breast cancer patients [29]), and only speculations can be made about the conformational modifications it might induce. Located between h9 and h10, mutation of this residue could notably affect ERα binding to heat shock proteins (HSPs) and/or dimer stability [32]. In addition to E380Q, the S463P mutation presents only slight constitutive activity assessed by target gene transcription, although no interaction with coactivators was detected without estrogen stimulation in vitro. Nonetheless, hormone-independent cell proliferation is observed when ERα contains this mutation, raising questions about its functional role in breast cancer [40].

It should be noted that several mutations are sometimes detected in the same tumor sample, but it could not be established whether they reside within the same cell population. Chandarlapaty et al., for example, identified concomitant of the D538G and Y537S mutations in the plasma of 30/541 patients (5.5%) with metastatic breast cancer [42]. In any case, this observation highlights the intrinsic heterogeneity of breast cancers and the mechanisms involved in the development of endocrine resistance [30].

Usually acquired after the first line of endocrine therapies, the mutations presented here counteract the efficacy of aromatase inhibitors and alter the inhibitory effect of SERMs and SERDs to various extents (Table 2).

According to in vitro studies, Y537S/N/C, D538G and L536Q mutations that confer ligand-independent activity to ERα remain sensitive to the SERM tamoxifen and the SERD fulvestrant when therapeutic doses are increased [34,35,37]. In contrast, in vitro and in vivo fulvestrant is effective in tumors driven by mutations such as E380Q and S463P, indicating the heterogeneity of therapeutic responses depending on the mutations driving breast cancer endocrine resistance [40]. From analysis of clinical data, it is unclear whether the SERD fulvestrant is effective for patients harboring LBD-activating mutations [31,51,52]. Using high doses of fulvestrant was then explored for patients developing resistance to aromatase inhibitors, and new therapeutic strategies were investigated to inhibit mutated ERα in a more potent and specific way. Notably, orally available SERDs with improved bioavailability are under development [53]. Additionally, Toy et al. showed by combining in vitro and in vivo studies that AZD9496 or GDC-0810 provided complete inhibition of tumors driven by mutated ERα, contrary to fulvestrant, against which the Y537S mutant was particularly resistant [40]. Likewise, elacestrant (RAD1901) inhibited ERα signaling and tumor development in PDX models harboring either wild type or mutant ERs [54]. Elacestrant is currently in a phase III trial for patients with ER-positive advanced breast cancer [55]. Patients with metastatic breast cancer carrying LBD *ESR1* mutations have poorer overall survival [31,42], which is consistent with the study by Jeselsohn et al. who showed that Y537S and D538G ERα mutants exhibit specific cistromes and transcriptomes that promote tumor metastatic phenotypes [43]. Thus, to treat cancers harboring *ESR1* LBD mutants, it will be necessary to develop combined treatments composed of SERD/SERM associated with other compounds, such as THZ1, a CDK7 inhibitor, making it possible to fight against the metastatic propensity of these mutants [43].

### 2.2. Outside the LBD

While less frequent than LBD-activating mutations, other ERα mutations are observed in breast cancer patients. The primary recurrent mutation is the K303R substitution, which was detected in 5–10% of invasive breast cancers, but could be more prevalent if more studies were performed with alternative sequencing techniques, according to Fuqua et al. [44]. Unlike mutations occurring in the LBD, the acquisition of this alteration in the hinge region does not appear to result from selection under endocrine therapy [44,45,46]. This part of the receptor is the target of multiple posttranslational modifications (PTMs): by affecting residues 266 to 305, modifications such as acetylation, phosphorylation, methylation, ubiquitination and sumoylation enable the interaction with multiple coregulators and play a role in DNA binding to regulate transcription [47].

The K303R mutation results in hypersensitivity to estrogen and a reduction in endocrine therapy efficacy due to a combination of molecular mechanisms that have been previously reviewed [44]. This phenotype is notably due to an increase in the phosphorylation of S305 by cellular kinases, such as PKA [48], and the inhibition of other PTMs surrounding the mutated residue. These modifications of the hinge region enhance the stability of the receptor, alter coregulator binding in favor of coactivator recruitment, and favor interactions with growth factor receptors and downstream signaling pathways. All of these factors lead to increased ligand-independent activity of ERα and a better response to estrogen stimulation, allowing tumor cells to grow in a low-estrogen environment, contributing to the resistance to aromatase inhibitors [49]. Additionally, the response to SERM is altered, and the mutated receptor responds to tamoxifen as an agonist [50], which is not the case for the SERD fulvestrant [48]. Structural data are lacking for our understanding of the phenotype caused by the K303R substitution in ERα. Considering the role played by the hinge region in protein interactions and PTMs of the receptor, such a mutation could lead to a conformational modification that would be relevant for endocrine resistance. Investigating the arrangement of ERα in response to the K303R mutation would then be of great interest to conceive new therapeutic strategies.

Other missense mutations were identified outside the LBD of ERα in patients with breast cancer, such as the S47T, N69K and A86V substitutions in the AF1 domain and the L296P point mutation in the hinge domain. Cell-based assays did not demonstrate alterations of their transcriptional activity compared to the wild type receptor, but no further studies were conducted to investigate these mutations or to decipher their potential role in breast carcinogenesis and drug resistance [56].

## 3. ERα Synonymous Mutations

Restriction fragment length polymorphisms (RFLPs) of the ERα gene were first identified in introns, as well as exons [57]. Subsequently, the development of DNA sequencing enabled the detection of additional silent mutations in breast cancer patients [58]. Many studies have investigated the association of such polymorphisms with the risk of developing breast cancer (Table 3).

Despite the number of studies realized, no consensus has emerged due to several drawbacks:

**Small sample size**: Most studies included only a few hundred patients and controls, resulting in low statistical power for determining associations;
**Control source**: Some studies compared data from breast cancer patients to controls originating from the entire population, whereas other studies used data from the hospital as a control, which could induce bias in the observed associations [59];
**Ethnicity**: Association studies are generally performed in populations from a unique geographical origin, resulting in conflicting results between women of European and Asian or African ancestry, for example, due to diverse genetic backgrounds [60,61];
**Analytic methods**: The heterogeneity of methods employed to analyze the association of *ESR1* silent mutations with breast cancer development plays a role in the inconsistency of conclusions as well.

Meta-analyses are then of interest to combine the results of several studies, enabling us to increase the sample size and gather data from different ethnic groups. According to the latest studies in this field, some *ESR1* variants effectively seem to be associated with breast cancer risk, such as rs2234693 and rs9340799, but data from patients of African ancestry or environmental factors were lacking in these analyses [62,63]. New association studies with larger sample sizes and more comprehensive information about patients’ lifestyles are needed to clarify the association of *ESR1* polymorphisms and synonymous mutations with breast cancer development to determine whether they are a relevant risk or prognostic factors.

Functional studies are also needed to decipher the role of synonymous ERα mutations in breast carcinogenesis. As they do not alter the sequence of the protein, experimental data are indeed lacking regarding the role that such mutations could play in ERα transcription, translation and functions.

**Figure 3:**
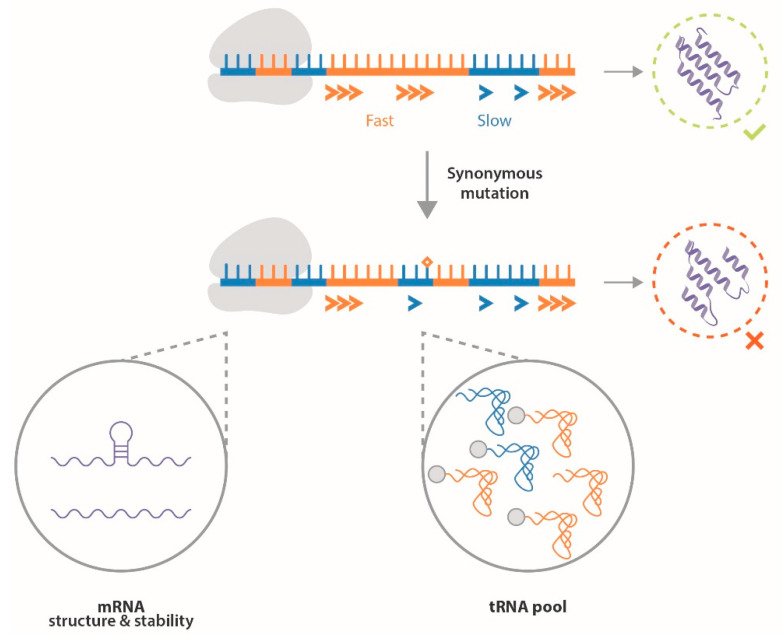
Synonymous mutations (represented by a star) can modify translational speed by impacting various parameters, such as the mRNA structure and the stability or availability of cognate tRNAs. This can ultimately result in the alteration of protein conformation, affecting its expression, stability and function.

After being ignored for a long time, synonymous mutations began to draw attention only more recently, following increasing evidence that codons used in mRNAs play a role in their translation. In fact, the use of synonymous codons can modify amino acid incorporation during translation elongation due to various parameters reviewed by Hanson and Coller [64]. First, the differential codon composition of an mRNA can alter its secondary structure and stability, which affects its translation rate [65,66,67]. Furthermore, the composition of the tRNA pool (concentration of each species, charging in amino acids, posttranscriptional modifications) results in differential availability of cognate tRNAs for each codon, which impacts thermodynamic parameters of anticodon-codon pairing and wobble base pairing [68]. This tRNA pool varies notably in a tissue-specific manner [69], depending on the differentiation state of the cell [70], and appears to be deregulated in cancer [71,72,73]. The combination of these factors can then alter the kinetics of translation during the initiation and elongation steps, which impact cotranslational folding of the emerging peptide. After being hypothesized in 1987 [74], it is indeed now admitted that many proteins begin to fold during their translation. This process already occurs in the polypeptide exit tunnel and when emerging from it through transient electrostatic interactions with the ribosome, protein folding activity of ribosomes (PFAR) and binding to chaperone proteins [68,75]. Translation speed is then a major actor of this cotranslational folding, with the use of codons translated faster or slower to characterize boundaries between protein domains, which participate in their proper folding [64,67,75]. Notably, the translational process is deregulated in cancer cells to meet their increased need for protein synthesis, enabling the expression of specific proteins for tumor growth [76]. Such a phenomenon notably relies on modifications of ribosome biogenesis in response to oncogenic signaling and is linked to the concept of specialized ribosomes [77,78]. This translational specificity of cancer cells could eventually participate in the modification of cotranslational folding of proteins as well. A mutation resulting in a synonymous codon substitution, although not modifying the amino acid sequence of the protein, can then alter mRNA stability and translation speed, resulting in the modification of protein folding and thereby altering protein expression, stability [79], and function [68,80]. In addition, the use of a synonymous codon less suited to the cellular environment can promote the incorporation of the wrong amino acid [67]. Therefore, synonymous mutations, although not modifying the sequence of the protein, appear to play a functional role in protein translation and function (Figure 3).

It is relevant to speculate that such mutations could play a role in the development of human diseases, as well as in the efficacy of therapies [68,75,81]. This began to be notably demonstrated with the example of the CFTR gene implicated in cystic fibrosis [82], or the oncogene KRAS [83]. With the development of next-generation sequencing and the increasing amount of data freely accessible through online databases, many more polymorphisms of the human genome could be investigated for their potential implication in human health [84].

Concerning ERα, it has been shown through in vitro translation that differences in the translation machinery modify the conformation of the produced receptor, which highlights a role of the translational process in the proper folding of the protein [85]. Fernández-Calero et al. showed that the synonymous mutation Ala87 (BstUI, rs746432) in the A/B domain of ERα alters its transcriptional activity and nuclear export, suggesting a conformational modification of the receptor [86]. Finally, Hertz and colleagues reported that another *ESR1* SNP (rs9322336) could be associated with an increase in ERα gene expression in ER-positive breast cancer patients, but no significant change of protein levels was recorded [87]. In light of recent research on cotranslational folding and the first data concerning ERα, we can hypothesize that synonymous mutations of this receptor could modify its conformation, playing a role in its interactions with cofactors and ligands, thereby altering its activity and cell fate regulation. Thus, further investigating the biological implications of synonymous ERα mutations through functional and structural studies would definitely be of great interest to increase our understanding of breast carcinogenesis and endocrine resistance and to identify new therapeutic strategies.

## 4. Conclusions

ERα is a major player in breast cancer development. By reducing circulating estrogen or directly inhibiting ERα, endocrine therapy is an effective strategy against luminal breast cancers. However, endocrine resistance exists in a significant number of patients, raising public health concerns. One of the mechanisms enabling tumor cells to escape hormonal control is mutation of ERα. Recurrent missense mutations were notably identified in the LBD of the receptor that confer ligand-independent activity by stabilizing its agonist form without estrogen binding. The development of biophysical studies has indeed enabled insights into the conformational modifications induced by ERα mutations detected in patients. Such information is of great value to better understand the biology of endocrine resistance and design new therapeutic strategies. In fact, the development of resistance towards standard of care highlights the need for more potent and selective agents against ERα in its native and mutated forms. In this regard, news SERDs such as GDC-0810, AZD9496 or RAD1901, were proven to efficiently target *ESR1* mutants in preclinical studies, with better pharmacokinetic properties than fulvestrant [40,54]. Furthermore, such therapeutic molecules can be used in combination with inhibiting actors of the metastatic process, such as cyclins, growth factors or PI3K/AKT/mTOR signaling pathways [88]. Novel molecules continue to show promising in vitro effects in recent years, such as AF2-specific inhibitors [89], selective ER covalent antagonist (SERCA) [90], and proteolysis-targeting chimera (PROTAC) [91]. In addition, the principle of biased ligands, well developed for GPCRs, could be applied to fine-tune ER signaling in breast cancer [92].

Structural data are still lacking for many point mutations known to play a role in hormonal escape of cancerous cells. The development of biophysical techniques should then improve the characterization of those receptors to develop more potent targeted therapies. A recent study conducted by Huang et al. revealed notable new interactions between the DBD and LBD of ERα that play a role in its genomic activity [93]. A missense mutation observed in endometrial cancer (Y191H) appeared to have a structural role in this interaction, which correlates with increased ERα transcriptional activity assessed in vitro [93]. While no oncogenic role has been reported for the Y191H substitution, this study highlights the significance of investigating ERα structure to extend our understanding of the mechanisms implicated in its activity and their potential roles in breast cancer development and endocrine resistance. Improving the detection of ERα mutations (notably by noninvasive methods) is critical as well to enable better patient care by adapting the therapeutic strategy faster in a personalized way [94,95]. In fact, the relevance of analyzing circulating DNA isolated from plasma samples to select therapy for breast cancer patients was recently assessed by Turner and colleagues [96].

In addition to point mutations leading to modifications of the amino acid sequence of ERα, synonymous mutations also exist. These mutations do not alter the protein sequence and were consequently ignored for a long time. Furthermore, no consensus has emerged regarding the association of such silent mutations with the risk of developing breast cancer. The association studies performed so far present several drawbacks, and more studies are needed to investigate the potential association of synonymous ERα mutations with breast cancer. Indeed, recent advances in the biology of codon usage and translation suggest that synonymous mutations play a role in a mechanism called cotranslational folding and therefore modify the conformation of the receptor. In this way, such mutations could modify ERα interactions and ligand binding, which could participate in tumorigenesis and endocrine resistance. More studies are needed to confirm whether synonymous mutations of ERα have a functional role in breast cancer development and therapeutic response.

Moreover, taking into account such a hypothesis would enlarge the field of therapies potentially suitable to overcome endocrine resistance. If synonymous mutations contribute to breast carcinogenesis by altering ERα function through the modification of its conformation due to an alteration of the cotranslational folding process, targeting the translational machinery could represent an additional line of treatment for endocrine therapy and the new ERα antagonists discussed above. With the increasing knowledge concerning the role played by ribosomes in tumorigenesis, this molecule has already emerged as a therapeutic target in cancer [97]. In addition, small molecules targeting the protein folding activity of ribosomes were developed as antiprion drugs [98], suggesting that more specific inhibitors of the ribosome could be designed as adjuvant therapies. In any case, investigating the role of synonymous mutations in human disease is a new area worth exploring [81,99].

## Figures and Tables

**Table 1:** Breast cancer classification based on receptor expression (ER: estrogen receptor; PR: progesterone receptor; HER2: human epidermal growth factor receptor 2).

Breast Cancer Type	Proportion	Biological Profile	Therapy of Choice
Luminal			Endocrine therapy
A	60%	ERα+ PR+/− HER2−	
B	10%	ERα+ PR+/− HER2+	
HER2-enriched [6]	20%	ERα+/− PR+/− HER2+	Anti-HER2 therapy
Triple negative [7]		ERα− PR− HER2−	Chemotherapy
Basal-like	7%	+ basal markers	
Non-basal-like	3%	− basal markers	

**Table 2:** Major ERα mutations discussed in this review and a summary of their characteristics. Purple: mutations in the LBD; blue: mutations outside the LBD (AI: aromatase inhibitor; SERD: selective estrogen receptor downregulator; SERM: selective estrogen receptor modulator; E2: estrogen).

ERα Substitution	Y537S/N/C	D538G	L536R/Q/P/H	E380Q	S463P	K303R
**Structural data obtained**	Stabilization of the agonist conformation	Stabilization of the agonist conformation				
**Ligand independent activity**	↑ target genes transcription ↑ coactivator recruitment ↑ proliferation	↑ target genes transcription ↑ coactivator recruitment ↑ proliferation ↑ migratory properties	↑ target genes transcription ↑ coactivator recruitment	↑ target genes transcription ↑ proliferation	↑ target genes transcription ↑ proliferation	↑ ERα stability ↑ coactivator recruitment ↑ interactions with growth factor receptors
**Estrogen and antiestrogen responses**	AI resistance ↓ SERD sensitivity SERM resistance	AI resistance SERD sensitivity SERM resistance	AI resistance SERD sensitivity	AI resistance SERD sensitivity SERM sensitivity ↑ E2 sensitivity	SERD sensitivity SERM sensitivity	AI resistance SERD sensitivity SERM = agonist activity ↑ E2 sensitivity
**References**	[26,30,31,32,33,34,35,36,38,40,42,43]	[26,30,31,32,33,34,35,37,40,42,43]	[32,34,40]	[30,31,32,38,39,40,41]	[32,35,40]	[44,45,46,47,48,49,50]

**Table 3:** Main polymorphisms of the ERα gene (*ESR1*) investigated in association studies. The major allele was selected in agreement with the single nucleotide polymorphism database dbSNP. (RFLP: restriction fragment length polymorphism).

RFLP	rsID	Domain	Codon	Major Allele	Minor Allele	Amino Acid
PvuII	rs2234693		397 (Intron 1)	T	C	
XbaI	rs9340799		351 (Intron 1)	A	G	
	rs2077647	A/B	10 (Exon 1)	TCT	TCC	Ser
BstUI	rs746432	A/B	87 (Exon 1)	GCG	GCC	Ala
		C	243 (Exon 3)	CGC	CGT	Arg
	rs1801132	E	325 (Exon 4)	CCG	CCC	Pro
	rs2228480		594 (Exon 8)	ACG	ACA	Thr

## Abbreviations

AF1	transcriptional activation function 1
DBD	DNA-binding domain
E2	estrogen
ERα	estrogen receptor alpha
ERBS	estrogen receptor binding site
ERE	estrogen responsive element
GF	growth factor
HER2	human epidermal growth factor receptor 2
HSP	heat shock protein
LBD	ligand-binding domain
LBP	ligand-binding pocket
PDX	patient derived xenograft
PFAR	protein folding activity of ribosomes
PR	progesterone receptor
PROTAC	proteolysis-targeting chimera
PTM	posttranslational modification
RFLP	restriction fragment length polymorphism
SERCA	selective ER covalent antagonist
SERD	selective estrogen receptor downregulator
SERM	selective estrogen receptor modulator
SRE	serum responsive element
TF	transcription factor
